# Conversion Disorder (Functional Neurological Symptom Disorder) Masquerading as Multiple Sclerosis: A Case Report

**DOI:** 10.7759/cureus.4893

**Published:** 2019-06-13

**Authors:** Derman Ozdemir, Sonu Sahni

**Affiliations:** 1 Internal Medicine, Saba University School of Medicine, Brooklyn, USA; 2 Internal Medicine, Brookdale University Hospital Medical Center, New York, USA

**Keywords:** functional neurological symptom disorder, conversion disorder, lhermitte’s sign, demyelination, shock-like sensation, dsm-5, fnsd, multiple sclerosis (ms)

## Abstract

Conversion disorder, also referred to as functional neurological symptom disorder, is a DSM-5 identified somatic disorder that presents with one or more neurological symptoms that does not clinically correlate with recognized neurological or medical conditions brought on by intense stress, emotions, or an associated psychiatric disorder. Multiple sclerosis (MS) is the most common immune-mediated inflammatory demyelinating disease of the central nervous system and usually presents in young adults with clinical manifestations that range from cognitive abnormalities, eye movement problems, motor and sensory impairments such as weakness and numbness, bowel/bladder dysfunction, fatigue, and/or pain. This case report presents a patient with functional neurological symptom disorder presenting with clinical signs associated with MS.

## Introduction

Conversion disorder, also known as functional neurological symptom disorder (FNSD), is a somatic disorder caused by severe stress, emotional conflict, or an associated psychiatric disorder usually presenting with one or more neurologic symptoms [1]. Conversion disorder is characterized by neurologic symptoms (e.g., weakness, abnormal movements, or nonepileptic seizures) that are inconsistent with a neurologic disease, but cause distress, and/or impairment [2]. The disorder is common in clinical settings and often has a poor prognosis [3-6]. A review found that the estimated incidence of conversion disorder across disparate geographical settings was four to 12 per 100,000 per year, and the community prevalence of conversion disorder based upon case registries was 50 per 100,000 per year [7]. Multiple sclerosis (MS) is a progressive degenerative disease of the central nervous system (CNS) whose symptoms and presentation depend on the severity of disease and the site of CNS lesions [8]. MS, an immune-mediated inflammatory disease, is the most common demyelinating disease of the CNS which often presents in a young adult with a clinically isolated syndrome such as optic neuritis, long tract symptoms/signs (e.g., numbness, paresthesia, or weakness), a brainstem syndrome (e.g., internuclear ophthalmoplegia), or a spinal cord syndrome (e.g., transverse myelitis) [9]. It is reported that MS may result from a combination of genetic, geographical, and that environmental factors such as vitamin D deficiency, season of birth, Epstein-Barr virus infection, and smoking, contribute to its incidence [10]. The authors report a patient that presented with common clinical signs and symptoms of MS but lacked clinical evidence to confirm the diagnosis and conversion disorder was diagnosed.

## Case presentation

**Figure 1:**
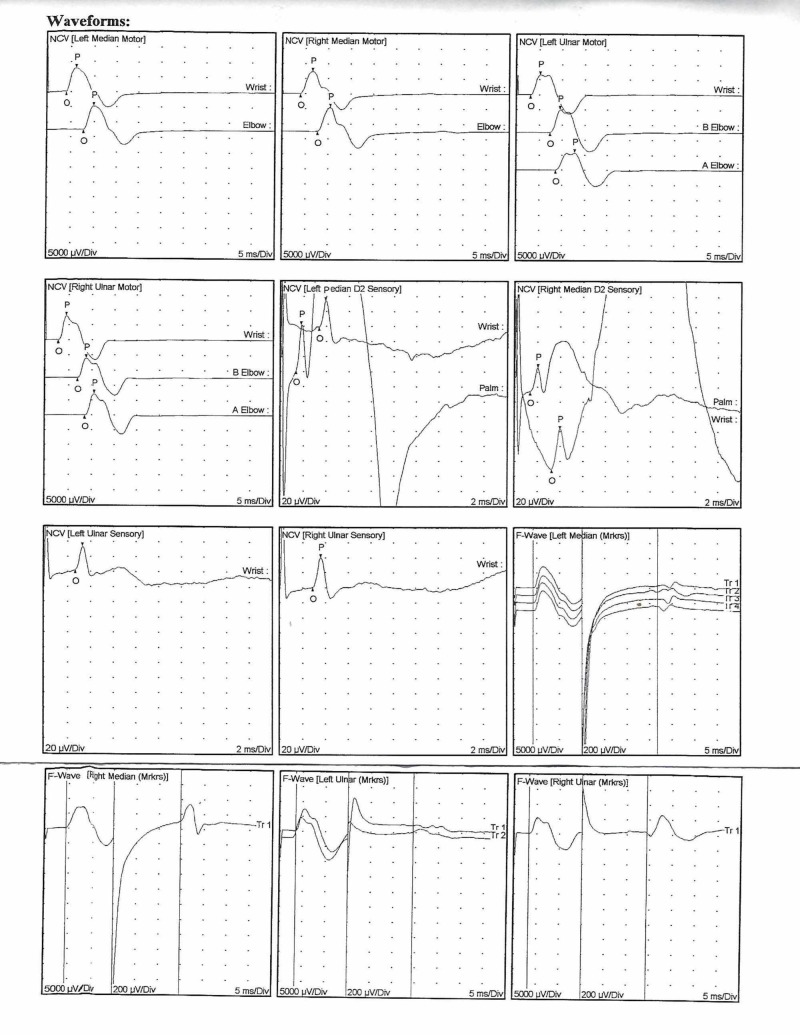
Nerve conduction study and F wave latency waveforms. Top row, in order from left to right: First waveform shows the motor nerve conduction of the left median nerve; Second waveform shows the motor nerve conduction of the right median nerve; Third waveform shows the motor nerve conduction of the left ulnar nerve. All waveforms are within normal limits with the exception of the borderline right and left median motor distal latencies are suspicious for mild, bilateral carpal tunnel syndromes. Second row, in order from left to right: First waveform shows the motor nerve conduction of the right ulnar nerve; Second waveform shows the sensory nerve conduction of the left median nerve; Third waveform shows the sensory nerve conduction of the right median nerve. All waveforms are within normal limits. Third row, in order from left to right: First waveform shows the sensory nerve conduction of the left ulnar nerve; Second waveform shows the sensory nerve conduction of the right ulnar nerve; Third waveform shows the F wave latency of the left median nerve. All waveforms are within normal limits. Fourth row, in order from left to right: First waveform shows the F wave latency of the right median nerve; Second waveform shows the F wave latency of the left ulnar nerve; Third waveform shows the F wave latency of the right ulnar nerve. All waveforms are within normal limits.

A 31-year-old right-handed Caucasian male student with no significant medical, psychiatric, or substance use history presented to a neurologist, from his primary care physician, complaining of bilateral paresthesia described as a shooting “pins-and-needles” sensation in the upper extremities, intermittent paralysis of the upper and lower extremities, daily fatigue, and an electric-shock like sensation that ran down the spine and extremities upon flexion of the neck (Lhermitte sign). The episodes were described by the patient as sudden onset with no relieving or exacerbating factors. The patient denied any recent weight change, vision disturbances, dizziness, nausea or vomiting. Outside of presenting symptoms, physical examination, blood, biochemistry, and thyroid function test results demonstrated no significant findings. The symptoms were considered to be due to carpal tunnel syndrome, however the use of wrist slings and rest, did not produce relief. The patient was reevaluated for neuropathic pain after a trial of gabapentin therapy (1200 mg/day divided into three doses for one week). It was found that the symptoms were unresponsive, at which point gabapentin was discontinued. Motor and sensory nerve conduction studies, used to derive the conduction velocity in the same segment of a limb (Table 1), and F wave latency, used to derive the conduction velocity of nerves between the limb and spinal cord (Table 2), were unremarkable. All examined muscles showed no evidence of electrical instability. Waveforms obtained from the observed conduction studies and F wave latencies were within normal limits except the borderline right and left median motor distal latencies which were concerning for mild, bilateral carpal tunnel syndromes but otherwise showed no consistent findings of MS (Figure 1).

**Table 1:** Nerve conduction studies. O-P: Onset-Peak; Amp: Amplitude; Vel: Velocity; Abd Poll Brev: Abductor Pollicis Brevis; Abd Digi Minimi: Abductor Digiti Minimi.

Motor Summary Table											
Site	NR	Onset (ms)	Normal Onset (ms)	O-P Amp (mV)	Norm O-P Amp	Site 1	Site 2	Delta-0 (ms)	Dist (cm)	Vel (m/s)	Normal Vel (m/s)
Left Median Motor (Abd Poll Brev) 32.5°C											
Wrist		3.9	<4	7.8	>5.0	Elbow	Wrist	3.8	21.5	57	>47.5
Elbow		7.7		7.7	>5.0						
Right Median Motor (Abd Poll Brev) 33.0°C											
Wrist		3.9	<4	7.2	>5.0	Elbow	Wrist	3.8	22	58	>47.5
Elbow		7.7		7.9	>5.0						
Left Ulnar Motor (Abd Digi Minimi) 32.5°C											
Wrist		3.0	<4	6.6	>3.0	B Elbow	Wrist	4.3	23.0	53	>47.5
Below Elbow		7.3		7.5	>3.0	A Elbow	B Elbow	1.3	9.5	73	>47.5
Above Elbow		8.6		5.7	>3.0						
Right Ulnar Motor (Abd Digi Minimi) 33.1°C											
Wrist		2.7	<4	7.9	>3.0	B Elbow	Wrist	4.2	25.0	60	>47.5
Below Elbow		6.9		6.4	>3.0	A Elbow	B Elbow	1.5	9.5	63	>47.5
Above Elbow		8.4		7.2	>3.0						
Sensory Summary Table											
Site	NR	Onset (ms)	Normal Onset (ms)	O-P Amp (mV)	Norm O-P Amp	Site 1	Site 2	Delta-0 (ms)	Dist (cm)	Vel (m/s)	Normal Vel (m/s)
Left Median D2 Sensory (2^nd^ digit) 32.9°C											
Wrist		3.4	<4	35.3	>15.0	Wrist	2^nd^ digit	3.4	15.0	44	
Palm		1.3		60.4	>15.0	Palm	2^nd^ digit	1.3	7.0	54	
						Wrist	Palm	2.1	8.0	38	
Right Median D2 Sensory (2^nd^ digit) 32.9°C											
Wrist		3.3	<4	52.7	>15.0	Wrist	2^nd^ digit	3.3	15.0	45	
Elbow		1.3		31.7	>15.0	Palm	2^nd^ digit	1.3	7.0	54	
						Wrist	Palm	2.0	8.0	40	
Left Ulnar Sensory (5th digit) 33.0°C											
Wrist		2.7	<4	30.9	>15.0	Wrist	5^th^ Digit	2.7	14.0	52	
Right Ulnar Sensory (5th digit) 33.0°C											
Wrist		2.9	<4	39.4	>15.0	Wrist	5^th^ Digit	2.9	14.0	48	

**Table 2:** F wave studies. F-Lat: F Latency; L-R: Left to Right; M-Lat: Motor Latency; Abd Poll Brev: Abductor Pollicis Brevis; Abd Digi Minimi: Abductor Digiti Minimi; Mrkrs: Markers.

Site	NR	F-Lat (ms)	F-Lat Normal (ms)	L-R F-Lat (ms)	L-R Lat Normal (ms)	M-Lat (ms)	FLat-MLat (ms)
Left Median Mrkrs (Abd Poll Brev) 32.4°C							
Wrist		31.76	<32.0	2.01	<2.0	4.15	27.61
Right Median Mrkrs (Abd Poll Brev) 33.0°C							
Wrist		29.75	<32.0	2.01	<2.0	4.29	25.46
Left Ulnar Mrkrs (Abd Digi Minimi) 32.9°C							
Wrist		29.41	<32.0	0.0	<2.0	3.46	25.95
Right Ulnar Mrkrs (Abd Digi Minimi) 33.0°C							
Wrist		29.41	<32.0	0.0	<2.0	3.18	26.23

**Figure 2:**
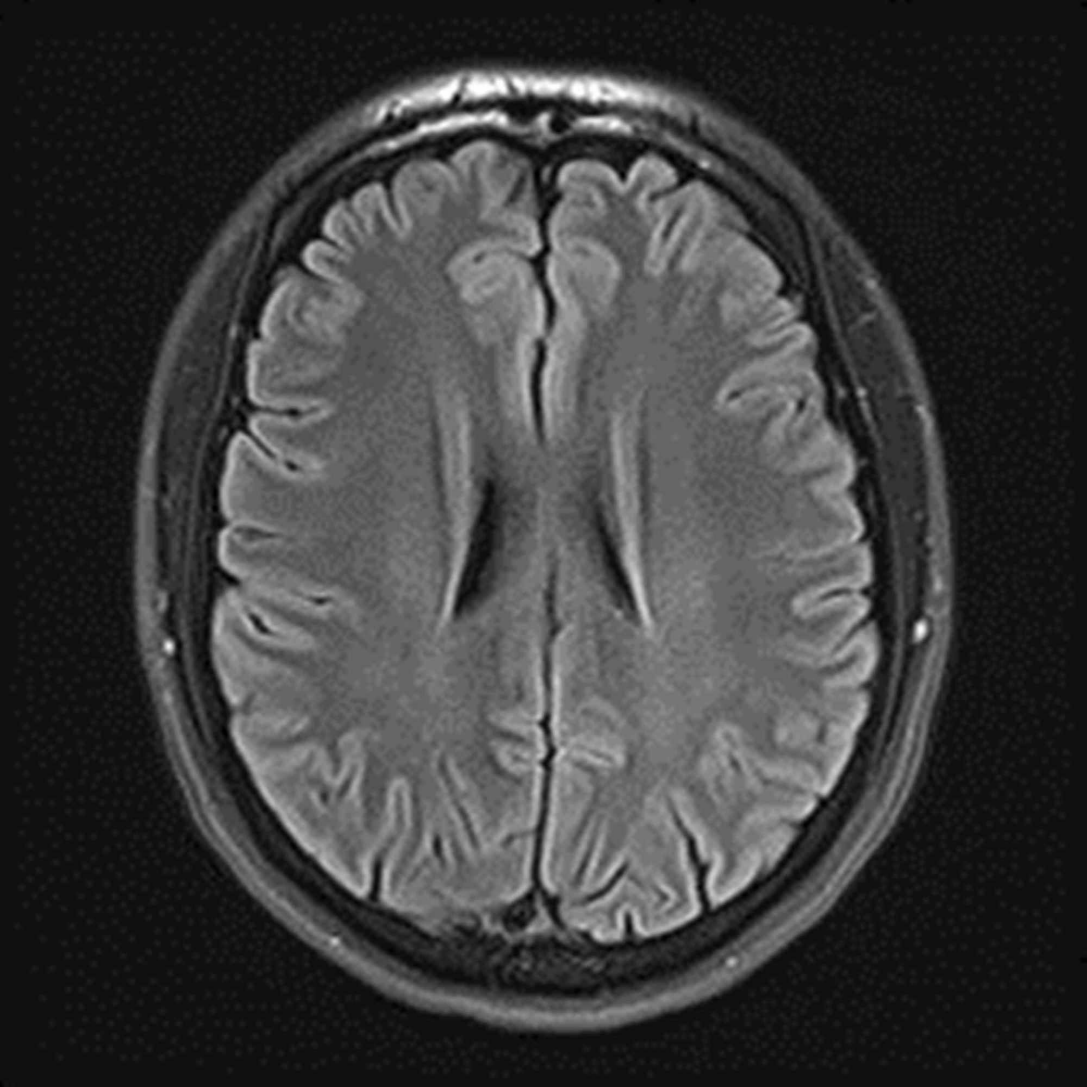
MRI-3T brain non-contrast showing normal brain with no evidence of demyelination.

**Figure 3:**
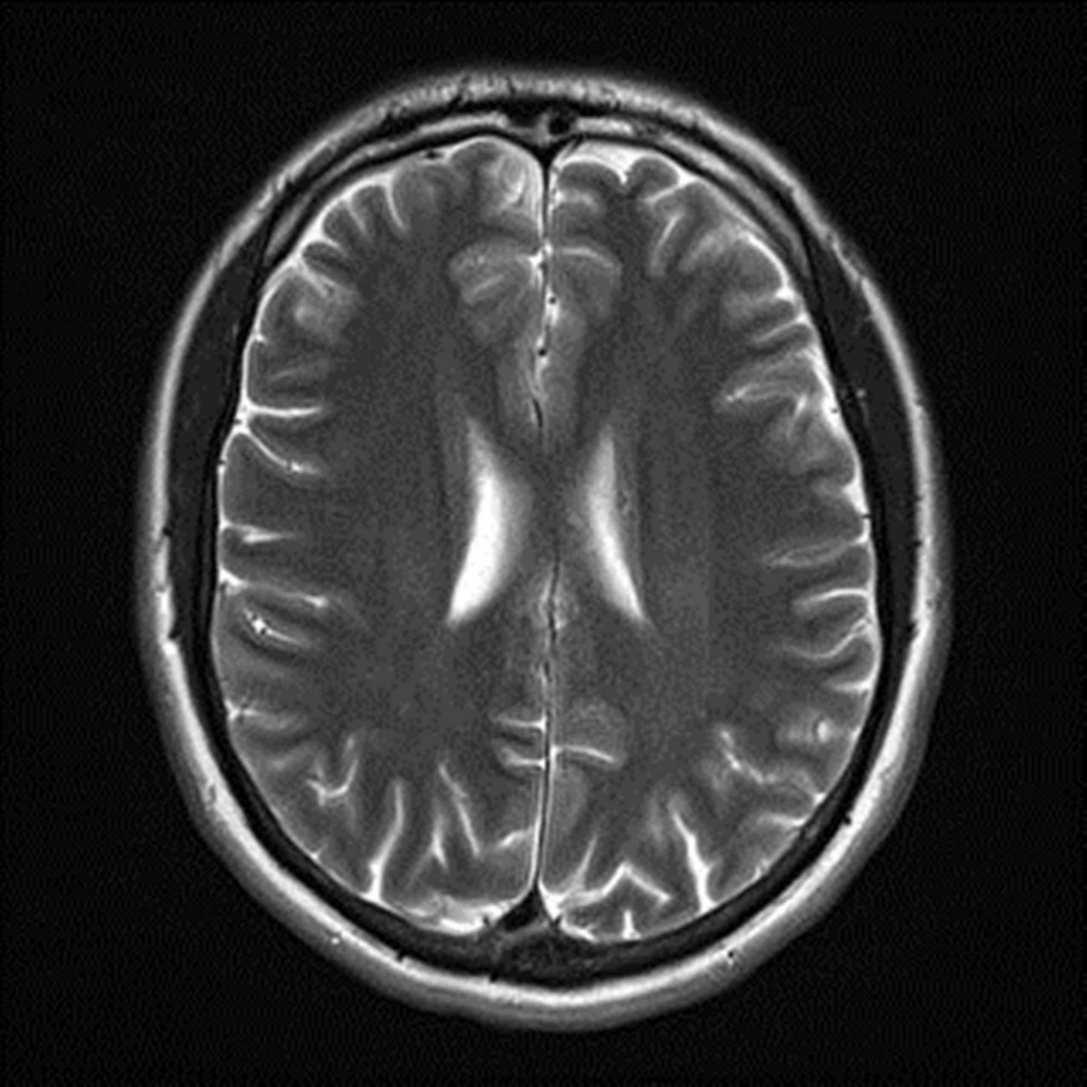
MRI-3T brain non-contrast showing normal brain with no evidence of demyelination.

The patient’s symptoms of extremity paresthesia and the presence of Lhermitte’s sign were suggestive of MS, so a magnetic resonance imaging (MRI-3T) of the brain without contrast was performed yielding unremarkable results that showed a normal brain with no evidence of demyelination (Figures 2, 3). Further assessment of the patient revealed that he had recently failed a class in school, which was causing him grief due to financial and academic repercussions. He also admitted to failing familial obligations and recent stress due to a personal relationship ending. The patient was asked of any previous seizure activity but denied any personal or family history of seizures, so an electroencephalography (EEG) was not ordered. The patient was advised to rest, reduce daily stress levels, and an explanation of conversion disorder, along with encouragement and reassurance was provided. It was explained that results did not correlate to a neurologic disease such as multiple sclerosis, to which the patient was receptive and understanding. At a one-month follow-up, the patient had re-enrolled in school, started exercising regularly, and reported decreased symptoms. Three-month follow-up showed that the patient had passed the initially failed class, was continuing a healthy lifestyle with exercise, had entered a new relationship, and no longer complained of any symptoms and/or reported any recurrences.

## Discussion

Herein we present a case of a 31-year-old male who presented with clinical symptoms consistent with MS. Multiple sclerosis typically presents in a young adult with a clinically isolated syndrome such as optic neuritis, long tract symptoms/signs (e.g., numbness, paresthesia, or weakness), a brainstem syndrome (e.g., internuclear ophthalmoplegia), or a spinal cord syndrome (e.g., transverse myelitis). Approximately 5 to 10% of adult patients have the primary progressive form of MS, which presents with gradual accumulation of disability from the onset, without superimposed acute relapses. The most common clinical presentation of primary progressive MS is a spinal cord syndrome with spastic paraparesis and no clear sensory level [11]. More common complaints of MS include sensory symptoms in limbs or face, visual loss, acute or subacute motor weakness, diplopia, gait disturbance, balance problems, and Lhermitte sign. First described by Marie and Chatelin in 1917 and named after Jean Lhermitte, Lhermitte's sign (also known as Lhermitte's phenomenon or the barber chair phenomenon) describes an electric shock-like sensation that occurs on flexion of the neck. MS patients describe the sensation as radiating down the spine, often into the legs, arms, and sometimes to the trunk as an electric shock like condition. The sensation occurs when the neck is moved in a wrong way or rather flexed. Demyelination and hyperexcitability are the main causes of Lhermitte's sign [12], along with vertigo, bladder problems, limb ataxia, acute transverse myelopathy, and pain. The onset is often polysymptomatic and the typical patient is a young adult with two or more clinically distinct episodes of central nervous system dysfunction with at least partial resolution with time. Proposed criteria for conversion disorder in the DSM-5 include "one or more symptoms of altered voluntary or sensory function" (Table 3 adapted from [13]). The clinical symptoms, "provide evidence of incompatibility between the symptom and recognized neurological or medical conditions," and "is not better explained by another medical or mental disorder" (Table 4). "The symptoms or deficit causes clinically significant distress or impairment in social, occupational, or other important areas of functioning or warrants medical evaluation" [14].

**Table 3:** Proposed criteria for conversion disorder in DSM-5.

Factor	Supporting History
Trauma/psychiatric symptoms	History of sexual abuse or trauma; increased stress; increased anxiety and panic symptoms; comorbid dissociative disorders
Somatic symptoms	Comorbid fatigue, chronic pain, irritable bowel syndrome; family concern over physical symptoms, exacerbating symptoms; impairment in sensory gating, allowing for excessive information loading
Illness exposure	Precipitating physical event or trauma; personal or family history of neurological disorder; personal or family history of other health disorder; profession in a medical field; media exposure to neurological disorder
Symptom monitoring	Impairment in habituation; increased focus on external body features; increased self-monitoring
Neuro-biological evidence	Abnormal attentional focus on affected area beliefs and expectations about illness deficits, in sense of control over actions; interregional neural network deficits in limbic system, sensorimotor areas and prefrontal cortex, functional and structural brain abnormalities

**Table 4:** Clinical features and assessment of conversion disorder (which may be episodic or acute or chronic).

Presenting symptoms
Non-epileptic seizures
Weakness and paralysis
Movement disorders
Speech disturbances
Globus sensation
Sensory complaints
Visual symptoms
Cognitive symptoms

Although the clinical manifestations of MS are variable, the most commonly quoted physical and cognitive effects of the disease include: weakness, fatigue, ataxia, bladder complaints, bowel problems, sensory effects and visual impairment [15-17]. Lhermitte’s sign has been seen to be very common in multiple sclerosis, but the prevalence of the symptom is unknown [8]. Lhermitte’s sign can also be seen with other lesions of the cervical cord, including tumors, cervical disc herniation, postradiation myelopathy, and trauma, none of which was seen in the presented patient. The most common initial feature of MS is the sensory symptoms which can reflect spinothalamic, posterior column, or dorsal root entry zone lesions. Symptoms are commonly described as numbness, tingling, pins-and-needles, tightness, coldness, or swelling of the limbs or trunk. Other clinical symptoms of MS which are more common due to the frequent occurrence of lesions, such as fatigue and motor symptoms that present as partial paralysis or paraplegia in extremities were also seen in our patient. The patient presented in this report complained of Lhermitte’s sign, without any predisposing factors, which has not been reported in patients with functional neurological symptom disorder. It may be infrequent or occur with the least movement of the head or neck. Furthermore, the patient also presented with other common clinical features of MS such as sensory symptoms, motor symptoms, and fatigue, which presented as bilateral paresthesias described as “pins-and-needles” sensation in the upper extremities, intermittent paralysis of the upper and lower extremities, and daily fatigue.

## Conclusions

In this case, the authors would like to point out that clinical features most commonly associated with multiple sclerosis (sensory and motor symptoms, fatigue, and Lhermitte’s sign) may not always be reflectively of MS, especially in the lack of predisposing factors. The diagnoses of MS remain a challenge due to the broad range of symptoms and clinical manifestations that might not be immediately evident. This case highlights the importance of a detailed patient history and evaluation of the psychosocial aspects of the patient. A patient who presents with symptoms of an organic neurological disorder such as MS, should be evaluated to rule out conversion disorder, which has previously not shown to exhibit the same clinical features as MS, as seen with the presented case.
